# Clinical Evaluation of QuickNavi^TM^-Ebola in the 2018 Outbreak of Ebola Virus Disease in the Democratic Republic of the Congo

**DOI:** 10.3390/v11070589

**Published:** 2019-06-28

**Authors:** Sheila Makiala, Daniel Mukadi, Anja De Weggheleire, Shino Muramatsu, Daisuke Kato, Koichi Inano, Fumio Gondaira, Masahiro Kajihara, Reiko Yoshida, Katendi Changula, Aaron Mweene, Placide Mbala-Kingebeni, Jean-Jacques Muyembe-Tamfum, Justin Masumu, Steve Ahuka, Ayato Takada

**Affiliations:** 1 Institut National de Recherche Biomédicale, Avenue de la Démocratie, Kinshasa/Gombe-P.O. Box 1197, Kinshasa I, Democratic Republic of the Congo; 2 Service de Microbiologie, Cliniques Universitaires de Kinshasa, Kinshasa, Democratic Republic of the Congo; 3 Department of Clinical Sciences, Institute of Tropical Medicine, Antwerp, Belgium; 4 Outbreak Research team, Institute of Tropical Medicine, Antwerp, Belgium; 5 Denka Seiken Co., Ltd., 1359-1, Kagamida, Kigoshi, Gosen, Niigata 959-1695, Japan; 6 Division of Global Epidemiology, Research Center for Zoonosis Control, Hokkaido University, Kita-20, Nishi-10, Kita-ku, Sapporo 001-0020, Japan; 7 School of Veterinary Medicine, the University of Zambia, Great East Road Campus, Lusaka, Zambia; 8 Africa Center of Excellence for Infectious Diseases of Humans and Animals, the University of Zambia, Lusaka, Zambia; 9 Global Station for Zoonosis Control, Global Institution for Collaborative Research and Education, Hokkaido University, Kita-20, Nishi-10, Kita-ku, Sapporo 001-0020, Japan

**Keywords:** Ebola virus, EVD, rapid diagnostic test, immunochromatography, QuickNavi, DRC

## Abstract

The recent large outbreaks of Ebola virus disease (EVD) in West Africa and the Democratic Republic of the Congo (DRC) have highlighted the need for rapid diagnostic tests to control this disease. In this study, we clinically evaluated a previously developed immunochromatography-based kit, QuickNavi^TM^-Ebola. During the 2018 outbreaks in DRC, 928 blood samples from EVD-suspected cases were tested with QuickNavi^TM^-Ebola and the WHO-approved GeneXpert. The sensitivity and specificity of QuickNavi^TM^-Ebola, estimated by comparing it to GeneXpert-confirmed cases, were 85% (68/80) and 99.8% (846/848), respectively. These results indicate the practical reliability of QuickNavi^TM^-Ebola for point-of-care diagnosis of EVD.

## 1. Introduction

Ebola virus (EBOV) is known to cause severe hemorrhagic fever in humans and/or nonhuman primates with human case fatality rates of up to 90% [1]. Five distinct ebolavirus species are known: *Zaire ebolavirus* (i.e., EBOV), *Sudan ebolavirus*, *Taï Forest ebolavirus*, *Bundibugyo ebolavirus*, and *Reston ebolavirus*. Ebola virus disease (EVD) poses a significant public health threat as shown by the largest EVD epidemic during the years 2013–2016 in West Africa. The second largest EVD outbreak is currently ongoing in the Democratic Republic of the Congo (DRC) where a cumulative total of 2181 confirmed and probable EVD cases and 1459 deaths have been reported since the beginning of the outbreak (as of 17 June 2019) [2]. These large-scale outbreaks emphasize the need for sensitive, easy-to-use, and robust rapid diagnostic tests (RDT) to enable quick and reliable screening of suspected EVD cases at the point-of-care. By facilitating early detection, RDTs can contribute to controlling the spread of the virus, especially in a context with known resistance to centralized EVD care.

The WHO-approved GeneXpert^®^ (Cepheid) technology, which is currently used in the field to diagnose EVD, is a major step forward compared to the conventional reverse transcription-PCR (RT-PCR) methods used previously in terms of turnaround time, ease-of-use and performance. However, this method still requires trained technicians and an uninterrupted power supply and is therefore considered a ‘near’ point-of-care test. Immunochromatography (IC)-based RDT assays for EVD could be a complementary first-line screening strategy at decentralized ‘points-of-care’ in the community.

We developed an IC-based RDT for EVD, QuickNavi^TM^-Ebola, using mouse monoclonal antibodies (mAbs) specific to the ebolavirus nucleoprotein (NP) [3]. This kit can detect ebolaviruses in the species *Zaire ebolavirus*, *Bundibugyo ebolavirus*, and *Taï Forest ebolavirus* with equally high sensitivity, but cannot differentiate these viruses. Since 2016, QuickNavi^TM^-Ebola has been continuously provided to the Institut National de Recherche Biomédicale (INRB), DRC, and experimentally used for early diagnosis of suspected EVD cases. It is particularly noted that QuickNavi^TM^-Ebola supported the diagnosis of initial EVD cases confirmed by INRB in May and August in the 2018 outbreaks in the DRC [4] (Table 1).

Currently, there are four RDTs approved by the FDA and/or WHO, ReEBOV (Corgenix), OraQuick Ebola (OraSure Technologies, Inc., Bethlehem, PA, USA), SD Q Line Ebola (SD Biosensor, Inc., Suwon, Korea), and DPP Ebola Antigen (Chembio, Medford, OR, USA) [5,6]. Of these, only OraQuick Ebola, which is based on the detection of the EBOV matrix protein, was available and used in the 2018 DRC outbreaks [4,7]. During the outbreak, QuickNavi^TM^-Ebola has also been experimentally used to assist screening of EVD-suspected patients at the outbreak sites. The RDT was used alongside confirmatory molecular testing, but its results were not used for clinical decision making. We report in this paper on the diagnostic performance of the QuickNavi^TM^-Ebola based on field data from the North-Kivu/Ituri EVD outbreak.

## 2. Materials and Methods

### 2.1. Devices

**Figure 1:**
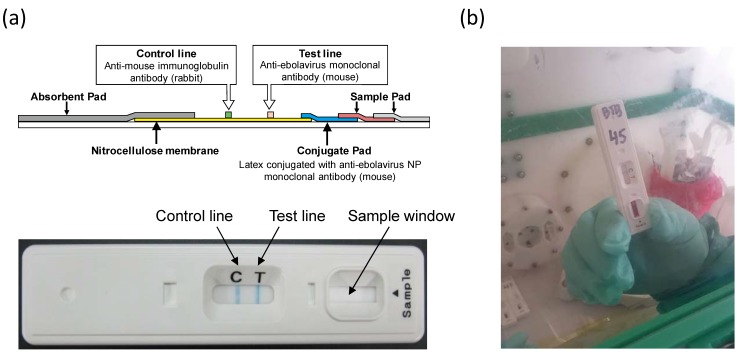
(**a**) Illustration of QuickNavi^TM^-Ebola. A sample added to the sample window of the device migrates via capillary action. The ebolavirus NP antigens present in the sample bind to the latex-conjugated mAb on the conjugate pad. Another mAb is immobilized on a nitrocellulose membrane at the Test line position and captures the complexes of NPs and mAbs conjugated with latex. Those complexes deposit a visible blue line. (**b**) QuickNavi^TM^-Ebola used at a field laboratory in North-Kivu province.

IC-based QuickNavi^TM^-Ebola devices were produced by using mouse mAbs ZNP105-7 and ZNP108-2-5 specific to the ebolavirus NP as described previously [3] (Figure 1a). Samples (10–30 μL) were applied onto the sample pad of the device, followed by the addition of 2 drops (approximately 40 μL) of the sample buffer supplied together with the devices. Results were interpreted 10–15 min later. Stability tests for QuickNavi^TM^ were performed according to a standard procedure in Denka Seiken Co., Ltd. The test kit devices were stored in a storage room kept at 33 ˚C. Using virus-like particles consisting of the viral glycoprotein (GP), viral protein 40 (VP40), and NP, performance data were periodically obtained at the specified time and the sensitivity and specificity in the experiment condition were monitored up to 24 months.

### 2.2. Realtime PCR

RealStar Filovirus Screen RT-PCR Kit (Altona Diagnostics, Hamburg, Germany), LightMix^®^ Ebola Zaire rRT-PCR Test (Roche Diagnostics, Basel, Switzerland), and GeneXpert^®^ (Cepheid, Sunnyvale, CA, USA) were performed according to the procedures provided by the manufacturer.

### 2.3. Sample Collection and Detection of the Virus

Blood samples obtained by venous blood draw were collected from suspected EVD patients at Ebola Treatment Centers or health facilities in North-Kivu province (Mangina and Beni) and Ituri province (Tchomia, Bunia, and Komanda) in the DRC and subjected to rapid screening with QuickNavi^TM^-Ebola followed by testing with RT-PCR-based detection of the viral RNA genome (i.e., GeneXpert approved by WHO). Both tests were performed in INRB-managed field laboratories linked to the above-mentioned health facilities (Figure 1b). Most of the samples were analyzed within 5 h after sample correction. The technicians applied strict biosafety measures and adhered to the manufacturers’ instructions. Blood collection and clinical evaluation during the outbreak investigations were approved as standard care by the Ministry of Health of the DRC and oral consent was obtained from all patients before blood sampling.

## 3. Results and Discussion

During the outbreak caused by an ebolavirus (*Zaire ebolavirus*) in 2018–2019 [8], 928 whole blood samples collected from suspected EVD cases were tested with QuickNavi^TM^-Ebola and the WHO-approved GeneXpert. Whenever QuickNavi^TM^ tests were available, they were used in a systematic manner on the first blood sample (and repeat if symptomatic less than 72 h at first blood draw) of each new EVD-suspect case. QuickNavi^TM^ testing was always performed and read before GeneXpert testing. The definition of positive and negative cases is as follows. Negative: GP not detected/NP not detected, GP not detected/CT of NP ≥ 40, or GP detected/NP not detected (considered negative or vaccinated case depending on vaccination history). Positive: GP detected/NP detected (CT < 40) or GP not detected/NP detected (CT < 40).

The sensitivity and specificity of the QuickNavi^TM^-Ebola assay were estimated by comparing its results to the GeneXpert-results and confidence intervals (CI) based on the F-distribution approximation were calculated (Table 2). Of the 80 GeneXpert-positive samples, 68 samples were positive with QuickNavi^TM^-Ebola, which represents a sensitivity of 85% (95% CI; 75.26–92.00). Most (10 of 12) were GeneXpert-positive, but QuickNavi^TM^-Ebola-negative samples had high CT values (30 <) both in NP and glycoprotein (GP) gene targets, and half of these samples were negative for detection of the GP gene (Table 3). Indeed, the distribution of CT values for NP gene detection indicated that the median, interquartile range, and mean of QuickNavi^TM^-Ebola-positive samples were remarkably lower than those of QuickNavi^TM^-Ebola-negative samples (Table 4). The QuickNavi^TM^-Ebola-negative samples that showed low CT values might result from a prozone effect or aggregation of too many antibody–antigen complexes which might restrict a flow on the membrane. Importantly, most of the GeneXpert-negative samples (846/848) were also negative with QuickNavi^TM^-Ebola, giving a specificity of 99.8% (95% CI; 99.15–99.97).

Considering that PCR-based assays generally show higher sensitivity than IC-based RDTs, it was quite reasonable that most samples false-negative with QuickNavi^TM^-Ebola showed relatively high CT values in real-time PCR-based GeneXpert (Table 4). In experimental conditions, the limit of detection (LOD) of QuickNavi^TM^-Ebola was 10^3^–10^4^ focus forming units/mL for infectious Ebola virus and 33 ng/mL for the purified recombinant EBOV NP [3]. According to the manufacturer, the LODs of FDA/WHO-approved OraQuick Ebola and ReEBOV were 1.64 × 10^6^ TCID_50_/mL and 10^6^ plaque forming units/mL, respectively, and their sensitivities estimated with whole blood clinical samples were 84% (95% CI: 63.9–95.5) (OraQuick Ebola) and 78–96% (ReEBOV), respectively [5,9]. QuickNavi^TM^-Ebola had 99.8% specificity in this study, whereas the reported specificities of OraQuick Ebola and ReEBOV were 98% and 73–91%, respectively. Taken together, these data may implicate at least a comparable or even better performance of QuickNavi^TM^-Ebola than OraQuick Ebola and ReEBOV, as indicated by its lower LOD, similar sensitivity, and higher specificity, although this comparison should ideally made using the same set of clinical samples (e.g., head-to-head comparison on stored samples). It is also worth noting that our ongoing study suggests that QuickNavi^TM^-Ebola has a shelflife of at least 24 months at room temperature.

Ebolavirus particles consist of seven structural proteins [1]. Of these, NP, VP 40, and GP are known to abundantly present in the viral particles. In general, viral envelope glycoproteins are antigenically variable and thus thought to be unsuitable for IC tests that require the capacity to widely detect virus variants. The four RDTs approved by the FDA and/or WHO (i.e., ReEBOV, OraQuick Ebola, SD Q Line Ebola, and DPP Ebola Antigen) have been designed to detect VP40 [5,6]. In contrast, we used NP-specific mAbs to produce QuickNavi^TM^-Ebola. The EBOV particle contains approximately 3200 NP molecules which form large complexes of the nucleocapsid consisting of the helical nucleoprotein–RNA complex [10,11]. The NP antigen appears to be one of the ideal targets for IC assays for EVD because of the presence of common epitopes among ebolavirus species, its strong antigenicity, and the large oligomeric structure of NP complexes providing multiple antibody binding sites, which may enhance the sensitivity of the test [12]. The use of mAbs is also one of the advantages of QuickNavi^TM^-Ebola since the antibodies can be stably produced and can avoid the use of live animals to produce polyclonal antisera.

IC-based RDTs are currently used for various viral diseases such as influenza, human adenovirus infection, and norovirus infection with significant reliability [13]. Currently available EVD RDTs cost around 10–20 US dollars/test, while some other commercialized RDTs based on the immunochromatography generally cost around 1–10 US dollars depending on their targeted diseases. Since the clinical manifestations of EVD are usually non-specific and similar to those of other infectious diseases present in EVD endemic areas, IC-based RDTs can be powerful tools for diagnosis even in remote areas in African countries. Since it is not predictable where and when EVD outbreaks will occur in the future, it is important to quickly discriminate between EVD and other viral diseases for early cases of suspected EVD, which may enable us to respond immediately to potential outbreaks, followed by initiating EVD-specific countermeasures once it is confirmed [14]. Given the simplicity of the procedure of QuickNavi^TM^-Ebola, it would also be of benefit if repeated testing were done for patients who were QuickNavi^TM^-Ebola-negative in initial screening since increased plasma viral loads in such patients should ensure the detection in a few days.

The present study demonstrates that QuickNavi^TM^-Ebola has good sensitivity and specificity in clinical field conditions. The results were similar to or tended to be even better than those obtained for other Ebola RDTs with WHO and/or FDA approval for emergency use. Owing to its significant practical utility, including simplicity, high stability, and the absence of requirements for special equipment and training, QuickNavi^TM^-Ebola is expected to be a useful tool for point-of-care screening of EVD.

## Figures and Tables

**Table 1:** Diagnosis of initial Ebola virus disease (EVD) cases in the two 2018 outbreaks in the Democratic Republic of the Congo (DRC).

Sample ID	Outbreaks (Month)	QuickNavi^TM^-Ebola	Real-Time PCR
18M-1	1st (May)	Negative	Negative^†^
18M-2	1st (May)	Positive	Positive (23.6/28.7)^†^
18M-3	1st (May)	Negative	Negative^†^
18M-4	1st (May)	Negative	Negative^†^
18M-5	1st (May)	Negative	Negative^†^
18M-6	1st (May)	Positive	Positive (22.3/22.3)^†^
18A-1	2nd (August)	Negative	Positive/Negative (39.6/NA)^‡^^§^
18A-2	2nd (August)	Negative	Negative^‡^
18A-3	2nd (August)	Positive	Positive (21.6/25.0)^‡^
18A-4	2nd (August)	Positive	Positive (28.3/30.7)^‡^
18A-5	2nd (August)	Negative	Negative^‡^
18A-6	2nd (August)	Positive	Positive (20.1/23.7)^‡^

^†^ RealStar Filovirus Screen RT-PCR Kit (Altona Diagnostics) and LightMix^®^ Ebola Zaire rRT-PCR Test (Roche Diagnostics). CT values of positive samples are indicated in parentheses (Altona/Roche). ^‡^ GeneXpert (Cepheid) to detect NP and GP genes. CT values of positive samples are indicated in parentheses (NP/GP). ^§^ Positive for NP, but negative for GP. NA: not applicable.

**Table 2:** Performance of QuickNavi^TM^-Ebola at the outbreak sites.

	GeneXpert Ebola		
	Positive	Negative	Total
QuickNavi^TM^-Ebola Positive	68	2	70
QuickNavi^TM^-Ebola Negative	12	846	858
Total	80	848	928

Sensitivity: 85% (68/80) (95% CI; 75.26–92.00); Specificity: 99.8% (846/848) (95% CI; 99.15–99.97); Negative predictive value: 98.6% (846/858); Positive predictive value: 97.1% (68/70); Agreement rate: 98.5% (914/928).

**Table 3:** Details of QuickNavi^TM^-Ebola-negative samples in PCR-confirmed cases.

Sample	QuickNavi^TM^-Ebola	GeneXpert Ebola			
		NP	CT (NP)	GP	CT (GP)
1	Negative	Positive	38.7	Negative	NA^†^
2	Negative	Positive	39.9	Negative	NA
3	Negative	Positive	38.1	Positive	40.2
4	Negative	Positive	38.7	Negative	NA
5	Negative	Positive	26.1	Positive	31.6
6	Negative	Positive	34.2	Positive	42.3
7	Negative	Positive	36.0	Positive	41.1
8	Negative	Positive	33.6	Positive	37.9
9	Negative	Positive	38.0	Negative	NA
10	Negative	Positive	36.8	Negative	NA
11	Negative	Positive	37.7	Negative	NA
12	Negative	Positive	13.9	Positive	19.1

^†^ NA: not applicable.

**Table 4:** Distribution CT values of QuickNavi^TM^-Ebola positive and negative samples.

Statistics	QuickNavi^TM^-Ebola Positive (*n* = 68)	QuickNavi^TM^-Ebola Negative (*n* = 12)
Minimum	14.20	13.90
25 percentile	17.98	34.05
Median	21.00	37.25
75 percentile	24.88	38.25
Maximum	35.60	39.90
Mean	21.76	34.31
Standard deviation	4.69	7.40
